# Healthcare students’ prevention training in a sanitary service: analysis of health education interventions in schools of the Grenoble academy

**DOI:** 10.1186/s12909-023-04235-y

**Published:** 2023-05-02

**Authors:** Marie Kuenemann, Mélanie Gaillet, Rebecca Shankland, Joey Fournier, Bastien Boussat, Patrice François

**Affiliations:** 1 Department of Epidemiology and Medical Evaluation, University Hospital of Grenoble-Alpes, Grenoble, France; 2 DIPHE, University Lumière Lyon 2, Lyon, France; 3 University Institute of France, Paris, France; 4 TIMC-IMAG Laboratory, University of Grenoble Alpes, Grenoble, France; 5 Service d’épidémiologie et évaluation médicale, CHU Grenoble-Alpes, Pavillon Taillefer, La Tronche, 38700 France

**Keywords:** Prevention training, Health promotion, Healthcare students, Health education, Sanitary service, Multidisciplinary training

## Abstract

**Background:**

The sanitary service is a mandatory prevention training programme for all French healthcare students. Students receive training and then have to design and carry out a prevention intervention with various populations. The aim of this study was to analyse the type of health education interventions carried out in schools by healthcare students from one university in order to describe the topics covered and the methods used.

**Method:**

The 2021–2022 sanitary service of University Grenoble Alpes involved students in maieutic, medicine, nursing, pharmacy and physiotherapy. The study focused on students who intervened in school contexts. The intervention reports written by the students were read doubly by independent evaluators. Information of interest was collected in a standardised form.

**Results:**

Out of the 752 students involved in the prevention training program, 616 (82%) were assigned to 86 schools, mostly primary schools (58%), and wrote 123 reports on their interventions. Each school hosted a median of 6 students from 3 different fields of study. The interventions involved 6853 pupils aged between 3 and 18 years. The students delivered a median of 5 health prevention sessions to each pupil group and spent a median of 25 h (IQR: 19–32) working on the intervention. The themes most frequently addressed were screen use (48%), nutrition (36%), sleep (25%), harassment (20%) and personal hygiene (15%). All students used interactive teaching methods such as workshops, group games or debates that was addressed to pupils’ psychosocial (mainly cognitive and social) competences. The themes and tools used differed according to the pupils’ grade levels.

**Conclusion:**

This study showed the feasibility of conducting health education and prevention activities in schools by healthcare students from five professional fields who had received appropriate training. The students were involved and creative, and they were focused on developing pupils’ psychosocial competences.

**Supplementary Information:**

The online version contains supplementary material available at 10.1186/s12909-023-04235-y.

## Background

Based on the observation that the French healthcare system is too focused on curative care, in 2017 the government drew up a national public health plan aimed at mobilising health care stakeholders on prevention activities. One part of this plan is the implementation of a training-action programme in prevention, called “sanitary service”, which is compulsory for all students in healthcare profession training.

Sanitary service was introduced during the 2018–2019 academic year, and is now part of the training curricula of healthcare students. Its objectives are: (1) to develop the students’ competence to carry out health promotion actions; (2) to ensure health promotion actions with various populations; (3) to encourage interprofessionality; (4) to learn how to manage a project in supervised autonomy. To this end, students are trained in the principles and techniques of health education and public health project management, and they must then develop and implement a health education intervention with a target audience.

When the sanitary service was set up, University Grenoble Alpes (UGA) developed a partnership with the Grenoble education authority [1] to organise student interventions in schools. The sanitary service was seen as an opportunity to expand health promotion activities, which are part of the national education system’s mission [2]. Compulsory school was viewed as an ideal setting for reaching all children, especially those farthest from the healthcare system or from precarious or vulnerable families [3].

In France, several public policies, particularly the “educational health pathway” and the “Health Promoting School” approach of the Ministry of Education, are based on the development of psychosocial competences. These skills are defined by the WHO as the cognitive, social and emotional resources of individuals that enable them to respond effectively to the demands and challenges of daily life [4]. Numerous studies have shown the importance of psychosocial competences from the earliest age in the development of children, their well-being and their health [5]. Other studies have reported the effectiveness of programmes to develop psychosocial competences in youth in reducing addictive behaviours, violence, mental health and sexual health problems, increasing well-being [6, 7], furthering success at school [8], facilitating labour market integration and reducing health inequalities [9].

Furthermore, it has been shown that social influence, a sense of belonging and the school climate are levers of effectiveness for these programmes with young people [10, 11]. Finally, collaboration between health and education professionals is an important criterion for the success of such programmes [12, 13].

During the first health service session organised by UGA in 2018–2019 [1], the students’ interventions were based on the Unplugged programme [14] which was then deployed in the academy with the help of the university’s social psychology teams. This 12-session health education programme for the prevention of addictive behaviours in schools [15], which has been validated and implemented in several European countries, is aimed at secondary school students. Since then, the organization of the sanitary service has gradually evolved so as to adapt to various constraints: the intervention model has been standardized, with the number of sessions reduced to five; the programme has been extended to primary schools and three nursing schools joined the programme in 2021, thereby doubling the number of students involved.

The aim of this study was to analyse the health education interventions carried out in schools in 2021–2022 by UGA sanitary service students, to describe the themes addressed and the educational methods used, and to verify the relevance of the training provided to the students in terms of their ability to develop and implement a public health project.

## Method

### Design of the study

This was a cross-sectional study aimed at describing the health education interventions carried out by UGA sanitary service students between November 2021 and January 2022 in schools in the Grenoble academy, which covered 5 departments with a population of 3.35 million and 619,719 pupils.

### The UGA sanitary service

The sanitary service involved students in medicine (3rd year), pharmacy (5th year), physiotherapy (4th year), maieutic (2nd year), and nursing (3rd year). Students received theoretical and practical training in health education methods.

Basic knowledge was provided through 20 to 30 h of online courses, in the form of commented slide shows divided into seven modules. Two modules introduced the determinants of health, the concepts of health education and the role of the school in health promotion. The other five modules dealt with specific prevention themes: addictive behaviors, nutrition and physical activity, sexual health, mental health and vaccinations.

For the practical training, a two-day seminar was organised. Derived from the Unplugged programme, the seminar presented, in an interactive mode, an approach to health education through the development of psychosocial competences. Students were invited to experiment with tools and animation activities that contribute to group reflection and exchange on a chosen prevention theme.

The students were divided into small groups (16 to 20) and supervised by two instructors. The instructors were healthcare professionals from the same five disciplines as the sanitary service students. The students were grouped according to their host structures. They were assigned to the placements according to an algorithm that took into account their wishes, the number of students desired by each structure, and their original field of study, with the aim of forming multi-professional groups.

At the end of the seminar, the students contacted the host establishment to discover the context, analyse the request and plan their intervention during November, December and January. The theme of the intervention was chosen according to the needs identified by the school, as perceived by the teachers, particularly the head teacher.

After having designed their sessions and had their intervention plan validated by the pedagogical referent, the students had to deliver the intervention in the school. One pair of students was responsible for conducting 5 interactive and progressive sessions with the same group of pupils (class or half of a class) under the supervision of a national education professional. These sessions were meant to contain “ice-breaker” activities (playful and collective activities aimed at energizing the group, improving its cohesion and generating a friendly climate), and interactive reflective activities such as workshops, group games or debates aimed at developing the pupils’ psychosocial competences. At the end of the sessions, the students had their interventions evaluated by the pupils. Each group of students assigned to the same school wrote an intervention report according to a predefined format and submit it to UGA for validation of the internship.

### Study population

The study focused on groups of students who intervened in schools (nursery schools, primary schools, secondary schools, or high schools). Student groups working in non-school settings (universities, associations, public services) were excluded from the study.

### Data collection

The intervention reports were read and analysed by two independent evaluators, MK and PF, epidemiologist physicians, and the information of interest was transcribed on a standardised form (see appendix).

General information included the composition of the student group, the school, the grade level involved and the number of pupils receiving the intervention. Information on the preparation of the intervention included analysis of the request, details of the literature search and the appropriateness of the intervention plan. Information on the content of the intervention included the theme of the intervention, ice-breaker activities and interactive reflective activities. Depending on the tools and activities used, the evaluators identified and rated the psychosocial competences mobilised among the relevant cognitive, social or emotional skills. Organisational information included the number and duration of health education sessions delivered for a class, and the time spent preparing the intervention, on transportation and on working with pupils. The presence of an evaluation of the intervention by the pupils and the results of this evaluation was recorded. After comparing the forms, discordances between evaluators were resolved by consensus after re-reading the report and the data were entered into an Excel file. This file did not contain any direct or indirect identifiers of pupils and schools.

### Statistical analysis

Qualitative variables were described by proportions and their 95% confidence intervals; quantitative variables by median and 25th-75th percentiles.

Associations between variables were analysed using the chi² test or Fisher’s exact probability test if necessary, for qualitative variables; Student’s t test or Wilcoxon non-parametric test, for quantitative variables. Missing data were not replaced. The threshold of statistical significance was set at 5%, in a two-sided situation.

Analyses were performed with Stata SE software (version 15.0 or later, StataCorp, College Station, TX, USA).

## Results

### Participants

**Fig. 1:**
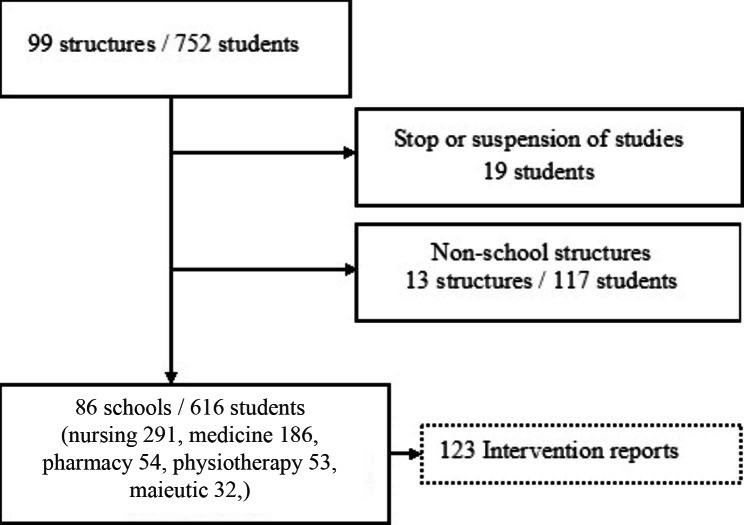
Study flow chart

Out of the 752 UGA healthcare students expected to perform sanitary service in 2021–2022, 19 stopped their studies during the year, and 117 were assigned to non-school institutions (Fig. 1). The study focused on the 616 students assigned to schools. They included 291 nursing students, 186 medical students, 54 pharmacy students, 53 physiotherapy students and 32 maieutic students. These students worked in 86 schools and submitted 123 intervention reports, which were analysed.

### Distribution of students in schools

The 86 schools were 17 nursery schools, 50 primary schools, 12 lower secondary schools and 7 upper secondary schools. The interventions involved 6853 pupils, the majority of whom (54%) were aged between 6 and 11 years (Table 1). The number of students assigned to a school ranged from 2 to 18, with a median of 6 students per site (IQR: 4–10). The 86 groups were composed of students from 1 to 5 streams with a median of 3 (IQR: 2–4) streams per group.

**Table 1:** Characteristics of schools and students involved in sanitary service, and students distribution

School (pupils’ age)	Pre-school (3 to 5 years)		Primary (6 to 11 years)		Secondary (12 to 15 years)		High school (16 to 18 years)		Total
	n	%	n	%	n	%	n	%	n
Number of schools (n, %)	17	20%	50	58%	12	14%	7	8%	86
Number of pupils involved (n, %)	889	13%	3695	54%	1410	21%	859	13%	6853
Number of students (n, %)	130	21%	326	53%	89	14%	71	12%	616
- including Nursing (n, %)	74	25%	157	54%	29	10%	31	11%	291
- including Medicine (n, %)	31	17%	100	54%	30	16%	25	13%	186
- including Pharmacy (n, %)	11	20%	28	52%	9	17%	6	11%	54
- including Kinesitherapy (n, %)	11	21%	26	49%	10	19%	6	11%	53
- including Maieutics (n, %)	3	9%	15	47%	11	34%	3	9%	32
Number of students per school (med, [IQR])	6 [4; 11]		6 [4; 8]		7 [4; 10]		10 [7; 12]		6 [4; 10]
Number of courses per school (med, [IQR])	3 [3; 4]		3 [2; 4]		4 [3; 4]		4 [3; 4]		3 [2; 4]

Med = median; IQR = Interquartile Range

### Intervention reports

The 86 student groups submitted 123 reports, written in groups or in pairs if the themes differed within the same school.

An analysis of the school’s request was included in all reports, a detailed description of the local context in 77% of the reports, and a description of the documentary research, most often via the Internet, on the theme and methods in 93% of the reports. The validation of the action plan by the group’s pedagogical referent was found in 87 reports (71%).

### Organisation of interventions

Most of the students (72%) conducted 5 health education sessions with the same group of pupils. Some teams had to cancel one or two planned sessions because classes were closed due to Covid-19 cases.

The time reported by the students for the intervention ranged from 19 to 39 h with a median of 25 h (IQR: 19–32) spent first on developing the intervention (median = 12 h, IQR: 8–20), then on working with the pupils (median = 5.5 h; IQR: 5-7.5) and transportation time (median = 4 h; IQR: 2.5-6).

### Intervention themes

The themes described in the reports were significantly different according to the age of the pupils (Table 2). The most frequent theme was “use of screens” (48% of the interventions) and concerned mainly primary schools (60%). This theme was often associated with “sleep hygiene”, which was addressed in 25% of cases. The second most frequent topic was “nutrition” (36%), which was proposed mainly in nursery schools (59%). The topic of “nutrition” was often associated with “physical activity”, which was mentioned by 9%. Interventions using Unplugged sessions were described in 25% of the reports, mainly in secondary schools (64%). Other themes mentioned included bullying (20% of reports), mainly in secondary schools, addictive behaviors (8%), and sexual health (6%).

**Table 2:** Themes (n = 123 reports)

	Pre-school n = 22 reports		Primary school n = 75 reports		Secondary school n = 14 reports		High school n = 12 reports		Total n = 123 reports		p^(1)^
	n	%	n	%	n	%	n	%	n	%	
Sceens (n, %)	4	18%	45	**60%**	6	43%	4	33%	59	48%	< 0.01
Nutrition (n, %)	13	**59%**	27	36%	2	14%	2	17%	44	36%	0.02
Sleep (n, %)	3	14%	27	**36%**	0	0%	1	8%	31	25%	< 0.01
Psychosocial competences (n, %)	4	18%	15	20%	9	**64%**	3	25%	31	25%	0.01
Harassment (n, %)	0	0%	14	19%	4	29%	7	**58%**	25	20%	< 0.01
Personal hygiene, -dental (n, %)	4	18%	14	19%	1	7%	0	0%	19	15%	0.38
Physical activity (n, %)	3	14%	7	9%	0	0%	1	8%	11	9%	0.61
Addictive behaviors (n, %)	0	0%	3	4%	4	**29%**	3	25%	10	8%	< 0.01
Sexual health (n, %)	0	0%	5	7%	1	7%	1	8%	7	6%	0.58
Domestic risks (n, %)	1	5%	3	4%	0	0%	0	0%	4	3%	0.99
Vaccination (n, %)	0	0%	1	1%	2	14%	0	0%	3	2%	0.10
Covid-19 (n, %)	0	0%	2	3%	0	0%	1	8%	3	2%	0.45
Other (n, %)	0	0%	2	3%	1	7%	0	0%	3	2%	0.54

^(1)^ Fisher’s exact test

### Pedagogical contents of the sessions

Most students (84% of the reports) started one or more sessions with an ice-breaker activity. The most widely used were based on individual expression of the mood of the day. All students used interactive teaching methods in each session. The methods applied during the seminar were found in 80% of the reports. The students also used other methods found on the internet or imagined by them. The creative effort of the students was rated as important in 43% of the reports, especially by the nursery school teams.

In the choice of tools, the activities requiring the most reflection and maturity were more often used with the older pupils (brainstorming, Delphi method, envelope game, mime games, role-playing, metaplan, blazon), while sensory experiences were more often carried out with the younger ones (tastings, recognition and manipulation of photos and labels, use of paints or glitter). In the different class levels, visual supports were used, whether they were graphic representations constructed by the pupils or video projections.

Regardless of pupils’ ages, the interventions were aimed at developing their psychosocial competences (Table 3), mainly cognitive competences (96%), and critical thinking (95%). Social skills were addressed in 79% of the reports, with a significantly different distribution according to the age of the pupils: communication was more often addressed among the younger pupils (< 12 years old), while resilience and empathy were addressed among the older pupils.

**Table 3:** Psychosocial competence development (n = 123 reports)

	Pre-school n = 22 reports		Primary school n = 75 reports		Secondary school n = 14 reports		High school n = 12 reports		Total n = 123 reports		p^(1)^
	n	%	n	%	n	%	n	%	n	%	
Cognitive competences (n, %)	21	95%	72	96%	13	93%	12	100%	118	**96%**	0.88
- Critical thinking (n, %)	16	94%	67	94%	12	92%	12	100%	107	**95%**	0.91
- Creative thinking (n, %)	1	6%	10	14%	3	23%	2	17%	16	14%	0.58
- Decision-making (n, %)	0	0%	5	7%	1	8%	2	17%	8	7%	0.35
Social competences (n, %)	14	64%	62	83%	11	79%	10	83%	97	79%	0.29
- Communication (n, %)	12	**60%**	55	**73%**	7	54%	4	33%	78	65%	0.04
- Cooperation (n, %)	0	0%	22	29%	4	31%	3	25%	29	24%	**0.02**
- Resisting social pressure (n, %)	0	0%	12	16%	5	**38%**	5	**42%**	22	18%	**< 0.01**
- Empathy (n, %)^(2)^	1	5%	8	11%	4	**31%**	5	**42%**	18	15%	**0.01**
Emotional competences (n, %)	6	27%	19	25%	6	43%	2	17%	33	27%	0.51
- Emotion identification (n, %)	6	27%	13	18%	4	31%	2	17%	25	21%	0.53
- Emotional regulation (n, %)	2	9%	10	14%	2	15%	0	0%	14	12%	0.64
- Stress management (n, %)	0	0%	3	4%	0	0%	0	0%	3	3%	**1.00**

^(1)^ Fisher’s exact test

### Evaluation

An evaluation of the intervention at the last session was found in 63% of the reports. The tools used differed from class to class. Pupils were evaluated using graphic tools (smiley faces, daisies, weather forecasts) in the pre-school and primary school classes, and by means of questionnaires in the secondary and high school classes. Most of the evaluations concerned pupils’ satisfaction (74%), especially in pre-schools. Knowledge acquisition was less often assessed (32%), slightly less often in pre-schools. The evaluation rarely (12%) focused on changes in representations or behaviors, most often in secondary and high schools (p = 0.04).

## Discussion

This study of the interventions carried out as part of the 2021–2022 sanitary service shows that healthcare students were able to develop and implement health education sessions based on an interactive pedagogy that mobilised pupils’ psychosocial competences.

The seminar programme for sanitary service students is based on the Unplugged programme, which has been implemented in several European countries [15]. This programme, based on the development of psychosocial competences, has been shown to be effective in promoting health and well-being. Psychosocial competences promote social adjustment and educational success, and help to prevent substance use, mental health problems, violent behaviour and risky sexual behaviour [6–8]. However, the programme used by the UGA sanitary service differs from the original programme by accommodating academic constraints (agendas, coordination of the different actors, distance) and pupils’ ages. The students mobilised their own creativity skills to construct sessions based on group animation methods.

It is known that effective interventions need to be long-term and multi-faceted, and that they must involve active and interactive pupil participation [16]. They should also use a variety of pedagogical tools, addressing both specific and general skills, and be delivered in supportive environments [10]. Discussed during the seminar, these concepts enabled the students to adapt the content of their sessions beyond the Unplugged sessions for secondary school students. For example, the students were able to use pedagogical resources found during online documentary research or during exchanges with national education professionals. All in all, while the interventions carried out in the framework of the sanitary service remain in line with the pedagogical approach proposed by the Unplugged programme, they deviate in terms of their duration and contents. It will be necessary to evaluate the effects of this type of programme on pupils in terms of the adoption of health-promoting behaviour.

Experiences of pupil health education by health students have been reported in the literature on various prevention themes: smoking, overweight and obesity [17–23]. The interventions of students, who are close in age, seem to be particularly effective with adolescents on subjects such as alcohol, drug and tobacco consumption. In experiments reported by Brinker et al. medical student interventions appeared to be effective in primary prevention of tobacco use, and in promoting smoking cessation [20–22]. In France, a health service study conducted by medical students reported positive effects in terms of knowledge, skills and behaviour on pupils and positive effects on the students themselves [23]. Another study conducted in three countries found a positive association between health behaviours and attitudes towards preventive counselling for all medical students [24]. In our sanitary service experience, students from five healthcare professions were trained at the same time and worked together in the schools. The multidisciplinarity of the UGA sanitary service has been praised as a strong and original point in a nationwide evaluation of French sanitary service [25]. This was previously viewed as the most satisfactory aspect by 85% of the students questioned at the end of the first sanitary service [1]. Other studies have shown the importance of interdisciplinary work not only in raising students’ awareness of prevention and public health issues [26], but also in favouring teamwork in the practice of the health professions [27, 28]. However, the integration of different health professions in the same seminar programme is not easy to achieve, as shown by the publications of other French sanitary service experiences where interventions were carried out by only one or two professions [23, 25, 26, 29]. The main challenge was to harmonise the academic agendas of the five professions so that students could be available at the same time for training and for interventions.

The main limitation of this descriptive study is that it could not measure the medium or long-term effects of the health education programme on the pupils or the healthcare students. There was no assessment of the pupils’ psychosocial competencies before and after the intervention. Another limitation is related to the method of data collection. The interpretation of some of the data in the student reports may have been somewhat subjective. Double independent reading with a third reading in case of discrepancies nonetheless ensured control of inter-operator variability. Finally, it involved students from only one university. The context in which the sanitary service is carried out can be very different from one university to another [23, 25, 26]. However, it seemed useful to report on an original experience in terms of its pedagogical model.

## Conclusion

This experience shows that it is possible to involve students from five different healthcare profession training courses in the same prevention programme in schools. The students were able to grasp the tools proposed during their theoretical and practical training and to adapt them. They were involved in the programme and were creative in developing and delivering health education sessions. Further studies are now needed to assess the effectiveness of the programme on the risk behaviours of pupils and on the professional representations and practices of the students.

## Electronic supplementary material

Below is the link to the electronic supplementary material.

Supplementary Material 1

## Data Availability

The datasets generated and/or analysed during the current study are available from the corresponding author on reasonable request.
