# Supplementary material for: Healthcare students’ prevention training in a sanitary service: analysis of health education interventions in schools of the Grenoble academy
Source: BMC Med Educ. 2023 May 2;23:302. doi: 10.1186/s12909-023-04235-y (PMC10152411; doi:10.1186/s12909-023-04235-y)
Supplement: Supplementary file 1 — Supplementary Material 1 [file 12909_2023_4235_MOESM1_ESM.docx]

Person in charge of the collection (initials):____ Code of intervention site: ______

**School**

School category: nursery school ⬜ ; primary ⬜ ; secondary ⬜ ; High ⬜ ;

Number of reports for the same institution: ____

**Information on each report; report n°: _________**

Number of students ____, (of which : Nursing.___ ; Medicine___ ; Pharma___ ; Kine___ ; Maïeut____)

Class level: Little section ⬜ ; Middle section ⬜ ; Upper section ⬜ ; CP ⬜ ; CE1 ⬜ ; CE2 ⬜ ; CM1 ⬜ ; CM2 ⬜ ; 6^e^ ⬜ ; 5^e^ ⬜ ; 4^e^ ⬜ ; 3^e^ ⬜ ; Second ⬜ ; Premiere ⬜ ; Final year ⬜ ;

Groups : entire classes ⬜ ; half-classes ⬜ ; Number of groups:_____

Of which number of mono-professional pairs :_________

**Project preparation**

Context analysis: Detailed ⬜ ; Partial ⬜ ; Not specified ⬜ ;

*Detailed = health education policy of the institution, current or planned programs*

Analysis of the request: Detailed ⬜ ; Partial ⬜ ; Not specified ⬜ ;

*Detailed = specific issues, priorities of the institution*

Presentation of the session calendar: yes ⬜ no ⬜

Literature review: yes ⬜ no ⬜ ; If yes: SESA documentation ⬜ ; websites ⬜ ;

Books ⬜ ; Expert ⬜ If expert, specify the profession: ___________________________________

Validation of the intervention plan by the pedagogical referent: yes ⬜ ; no ⬜ ; no trace ⬜ ;

**Intervention**

**Themes:** Different themes according to grade: yes ⬜ no ⬜ ; not applicable ⬜

Addictions ⬜ ; Harassment ⬜ ; Screen use ⬜ ; Sleep ⬜ ; Nutrition ⬜ ; Physical activity ⬜ ; COVID ⬜ ; Sexual health ⬜ ; Vaccinations ⬜ ; Psychosocial skills ⬜ ; other ⬜ ;

If other, specify:_________________________________________________

**Tools, activities:** Inclusion in a current Unplugged programme: yes ⬜ no ⬜

Cohesion tools / Icebreakers: yes ⬜ no ⬜

If yes: Cross presentation ⬜ ; Personal weather ⬜ ; Interactive icebreaker (e.g. my name is and I like; find someone who) ⬜ ; Charter of the group ⬜ ; Balloon/Ball ⬜ ; Song ⬜ ; Groupings ⬜ ; Ostend tree and its variants ⬜ ; Other ⬜

If other, specify: ___________________________________________________

Tools for reflection (representations, ideas, opinions, debates) :

Activities from the seminar or techniques manual: yes ⬜ no ⬜ : Brainstorming ⬜ ; Metaplan ⬜ ; Line game ⬜ ; Envelope game ⬜ ; Abacus ⬜ ; The Blazon ⬜ ; Photo expression ⬜ ; Delphi ⬜ ; Role-playing (role-playing, scenario) ⬜ ; About the word ⬜ ; Unplugged sessions ⬜ ; Mime ⬜ ; Other ⬜

_____________________________________________________________________________________

Other activities: Questionnaire ⬜ ; Quizz ⬜ ; Video ⬜ ; Images or stickers ⬜ ; Cuttings ⬜ ;

Drawing/colouring ⬜ ; Graphic representation (map, pyramid, plate, flower, poster) ⬜ ; Puzzle ⬜ ;

Goose game ⬜ ; Book/History ⬜ ; Challenge ⬜ ; Other ⬜

If other technique, specify: ______________________________________________________________

______________________________________________________________________________________

Creative effort in choosing tools: important ⬜ ; medium ⬜ ; low ⬜ ; none ⬜ ;

**Psychosocial skills mobilised :**

Social skills yes ⬜ no ⬜ ; (including verbal and non-verbal communication ⬜ ; empathy ⬜ ; cooperation ⬜ ; resistance and negotiation skills ⬜ ; advocacy ⬜)

Cognitive skills yes ⬜ no ⬜ ; (including Decision-making ⬜ ; critical thinking and self-evaluation ⬜ ; creative thinking ⬜)

Emotional skills yes ⬜ no ⬜ ; (including Emotional regulation ⬜ ; stress management ⬜ ; self-evaluation and self-regulation ⬜)

**Summary**

Number of sessions per student (binomial or group) :____ : mean duration of a session :_____

*NB: if variable, give the average number of sessions per student (group)*

Number of children involved (estimated):____________

Estimated time spent (in hours per student) For transport:________

For preparation and coordination: ______

For action with the public:______

*NB: if variable, give mean time per student*

**Evaluation by students:** evaluation at each session: yes ⬜ no ⬜ ; final evaluation yes ⬜ no ⬜

If final evaluation; Informal oral evaluation ⬜ ; Formal evaluation ⬜ ;

Evaluation technique: Questionnaire ⬜ ; Head/Heart/Feet ⬜ ; Personal weather ⬜ ; Marguerite ⬜ ; Abacus ⬜ ; Other ⬜

If other technique, specify: ___________________________________________________

Evaluation topics: Public satisfaction ⬜ ; knowledge acquisition ⬜ ; change in representation ⬜ ; behavioural change ⬜ ;

Submission of quantitative results: Yes ⬜ ; partially ⬜ ; No ⬜ ;
